# Web‐based integrated bipolar parenting intervention for parents with bipolar disorder: a randomised controlled pilot trial

**DOI:** 10.1111/jcpp.12745

**Published:** 2017-05-16

**Authors:** Steven H. Jones, Jelena Jovanoska, Rachel Calam, Laura D. Wainwright, Helen Vincent, Ozgur Asar, Peter J. Diggle, Rob Parker, Rita Long, Matthew Sanders, Fiona Lobban

**Affiliations:** ^1^ Spectrum Centre for Mental Health Research Faculty of Health and Medicine Lancaster University Lancaster UK; ^2^ Division of Psychology and Mental Health The University of Manchester Manchester UK; ^3^ Department of Biostatistics and Medical Informatics Acibadem University Istanbul Turkey; ^4^ CHICAS Faculty of Health and Medicine Lancaster University Lancaster UK; ^5^ Parenting and Family Support Centre University of Queensland Brisbane Qld Australia

**Keywords:** Bipolar disorder, web‐based intervention, parenting intervention, parents, randomised controlled trial

## Abstract

**Background:**

People with bipolar disorder (BD) experience additional parenting challenges associated with mood driven fluctuations in communication, impulse control and motivation. This paper describes a novel web‐based self‐management approach (Integrated Bipolar Parenting Intervention; IBPI) to support parents with BD.

**Method:**

Parents with BD with children aged 3–10 years randomised to IBPI plus treatment as usual (TAU) or waitlist control (WL). IBPI offered 16 weeks access to interactive self‐management information concerning BD and parenting issues. Feasibility was through recruitment, retention and web usage. Clinical outcomes were assessed at baseline, 16, 24, 36 and 48 weeks. Trial Registration Number: ISRCTN75279027.

**Results:**

Ninety seven participants were recruited with 98% retention to end of intervention and 90% to final follow‐up (56%–94% data analysed of retained participants; higher rates for observer measures). 77% of IBPI participants accessed the website (53% accessed parenting modules). Child behaviour, parenting sense of competence and parenting stress improved significantly in IBPI compared to WL to end of intervention, sustained to 48 weeks. Impacts of IBPI on family functioning, parent mood and time to mood relapse were not significant.

**Conclusions:**

Online self‐management support for parents with BD is feasible, with promising improvements in parenting and child behaviour outcomes. A definitive clinical and cost‐effectiveness trial is required to confirm and extend these findings.

## Introduction

Bipolar disorder (BD) is an enduring mental health problem characterised by severe fluctuations in affective, cognitive and physiological functioning. BD impacts negatively on, sleep/wake cycles, impulse control, communication and motivation, which may undermine adaptive parenting (Phelan, Lee, Howe, & Walter, 2006; Vance, Jones, Espie, Bentall, & Tai, 2008); whilst mood variation can trigger inconsistent parenting (David, Styron, & Davidson, 2011; Dolman, Jones, & Howard, 2013). Support for parents with BD is particularly important as their children are at high‐risk for psychiatric conditions, including attention deficit hyperactive disorder (ADHD), depression, anxiety, substance abuse, sleep disorders and BD (Axelson et al., 2015; Duffy, Jones, Goodday, & Bentall, 2016; Mesman, Nolen, Reichart, Wals, & Hillegers, 2013). Despite this, few of these children receive clinical support (Calam, Jones, Sanders, Dempsey, & Sadhnani, 2012; Jones, Tai, Evershed, Knowles, & Bentall, 2006) which risks more severe psychological disorders in later life (Duffy et al., 2016). Parenting programmes offer a means to support parents in encouraging desirable behaviours in their children: consequent reductions in parenting stress might have secondary clinical benefits for parents.

Parenting programmes are effective in reducing child behaviour problems, including ADHD, conduct disorder and antisocial behaviour (NICE, 2013). Programmes such as the Triple P Positive Parenting Programme (Sanders, 2012), foster adaptive parenting through information and support based on social learning and cognitive behavioural principles. Triple P is effective in improving child behaviour problems, parenting skills (Sanders, Kirby, Tellegen, & Day, 2014; and also maternal mood (Sanders & McFarland, 2000).

However, the impact of general parenting interventions for parents with severe mental illness is unclear (Bee et al., 2014) because of the low quality of current evidence (Schrank, Moran, Borghi, & Priebe, 2015). Parents with BD support the idea of parenting self‐management interventions (Jones et al., 2014) but most do not access these, due to lack of availability and reluctance to acknowledge parenting issues to mental health services due to stigma and fear of over‐monitoring (Dolman et al., 2013).

Online interventions have potential to offer destigmatising support at times and in locations convenient to parents, at low cost (Kazdin & Blase, 2011). Supported online psychological interventions can improve clinical outcomes in mothers with postnatal depression (Kaplan, Solomon, Salzer, & Brusilovskiy, 2014; O'Mahen et al., 2014) and parenting in parents with and without mental health problems (Love et al., 2016; Sanders, Baker, & Turner, 2012; Sanders, Dittman, Farruggia, & Keown, 2014). Additionally, three studies of online Triple P indicated child behaviour improvements (Love et al., 2016; Sanders et al., 2012; Sanders, Dittman et al., 2014).

The potential benefits of online parenting approaches for parents with BD require further investigation. Our team reported on a pilot RCT combining online psychoeducational information on BD, with an online version of Triple P (Jones et al., 2014). Compared to waitlist control, intervention parents reported improved parenting skills and child behaviour. However, lack of follow‐up, lack of interactivity and comprehensiveness in the intervention and lack of assessment of impact on parental mood symptoms were study limitations. The current study addresses these to assess the feasibility, acceptability and potential clinical benefit of delivering an integrated bipolar parenting intervention (IBPI), using an interactive self‐management site for BD developed by our team, combined with Triple P online (Turner & Sanders, 2011) in an RCT design with follow‐up to 48 weeks postbaseline.

## Methods

### Study design and participants

A rater‐blind randomised controlled trial (preregistered: http://www.isrctn.com; ISRCTN75279027: UK NHS Ethics Committee approved; REC ref, 12/NW/7049) compared 16 weeks of parent access to IBPI plus treatment as usual (TAU) with wait list control (WL). Protocol is published (Jones et al., 2015).

Recruitment was from 17 UK NHS Trusts (30/1/13–16/4/14) through clinical and self‐referral. Inclusion criteria: (a) BD diagnosis confirmed by structured clinical interview (First, Gibbon, Spitzer, Williams, & Benjamin, 1997); (b) ≥1 child aged 3–10 years (consistent with the age range that the Triple P intervention was originally designed for; Turner & Sanders, 2011) with whom ≥10 hr of face‐to‐face contact weekly and (c) internet access and ability to provide informed consent. Exclusion criteria: (a) primary diagnosis of alcohol/other substance misuse; (b) already receiving a parenting intervention and/or intensive psychotherapy; (c) index child in receipt of structured psychological therapy and/or subject to child protection proceedings.

### Procedure and intervention

Potential participants completed a telephone prescreen. Following consent, BD diagnosis was confirmed using the Structured Clinical Interview for DSM IV Axis 1 disorders (SCID; First et al., 1997) and baseline assessments completed. Individual randomisation was conducted by MAHSC Trials Coordination Unit, Manchester which provided anonymised condition allocation information to RP who was not involved in any assessments. All assessment researchers were masked to treatment condition. Participants were reminded prior to each assessment to refrain from disclosing treatment allocation. All self‐report measures were completed online. Observer rated measures were completed in telephone interviews with the researchers.

### Integrated bipolar parenting intervention

Integrated Bipolar Parenting Intervention was developed with service users with BD and parenting experience (Jones et al., 2015). It included an eight module self‐management intervention with strategies for BD in parents covering the following: What is Bipolar Disorder?; Benefits and Challenges; Managing Emotions; Knowing Yourself; Mood Monitoring; Playing to your strengths; Planning for yourself; Finding support and final thoughts. Participants also received access to the eight module Triple P online intervention (Turner & Sanders, 2011) covering: What is positive parenting?; Encouraging desirable behaviours; Teaching new skills and behaviour; Managing misbehaviour; Dealing with disobedience; Preventing problems by planning ahead; Making shopping fun; Raising confident, competent kids. Both elements provided information and opportunities for reflection through interactive and multimedia features, including video clips, collaborative exercises and self‐evaluation. IBPI employed a normalising, self‐regulatory focus to avoid stigmatising or blaming participants. Participants could work through each module of the intervention in around 30 min. Intervention access period was 16 weeks.

### Assessment of outcomes

#### Feasibility and acceptability outcomes

Feasibility was evaluated through recruitment to target, retention to follow‐up in both trial arms and absence of adverse events associated with IBPI. Acceptability was measured through parent's adherence to, and completion of the intervention, through website usage data.

Follow‐up period was 48 weeks from randomisation (see Table 1) with blind assessments at baseline, 16 (end of intervention), 24, 36 and 48 week follow‐ups. All observer‐rated follow‐up assessments were completed via telephone. All self‐report assessments were completed online.

**Table 1:** Schedule of quantitative assessments

Assessment	Baseline	16 weeks	24 weeks	36 weeks	48 weeks
Primary clinical outcomes					
Strengths & Difficulties Questionnaire (SDQ)	✓	✓	✓	✓	✓
Eyberg Child Behaviour Inventory (ECBI)	✓	✓	✓	✓	✓
Parenting Scale (PS3)	✓	✓	✓	✓	✓
Parenting Sense of Competency Scale (PSOC)	✓	✓	✓	✓	✓
Parenting Stress Index (PSI)	✓	✓	✓	✓	✓
Secondary clinical outcomes					
Internal States Scale (ISS)	✓	✓	✓	✓	✓
Centre for Epidemiologic Studies Depression Scale (CES‐D)	✓	✓	✓	✓	✓
Altman Self‐Rating Mania Scale (ASRM)	✓	✓	✓	✓	✓
Confusion, Hubbub and Order Scale (CHAOS)	✓	✓	✓	✓	✓
SCID Life^a^	✓		✓		✓
Hamilton Depression Rating Scale (HAM‐D)^a^	✓		✓		✓
Bech‐Rafaelsen Mania Scale (MAS)^a^	✓		✓		✓

a Completed via telephone.

##### Parenting and child behaviour outcomes

Child behaviour problems were assessed using the Strengths and Difficulties Questionnaire (SDQ; Goodman, 2001) and the Eyberg Child Behavior Inventory (ECBI; Eyberg & Pincus, 1999). Parenting skills, confidence and stress were measured with the Parenting Scale (PS3; Arnold, O'Leary, Wolff, & Acker, 1993), the Parenting Sense of Competency Scale (PSOC; Johnston & Mash, 1989) and the Parenting Stress Index (PSI; Abidin, 1990) respectively.

##### Parental mood, relapse and family coherence outcomes

Time to bipolar relapse was measured using the SCID Longitudinal Interval Follow‐up Evaluation (SCID‐LIFE; Keller et al., 1987), whilst observer‐rated mania and depression were assessed by Hamilton Depression Rating Scale (HAM‐D; Hamilton, 1960) and Bech‐Rafaelsen Mania Scale (MAS; Bech, 2012; Bech, Rafaelsen, Kramp, & Bolwig, 1978). Self‐reported mood symptoms were measured by the Internal States Scale (ISS; Bauer et al., 1991), Centre for Epidemiologic Studies Depression Scale (CES‐D; Radloff, 1991) and Altman Rating Scale (ASRM; Altman, Hedeker, Peterson, & Davis, 1997). Family coherence and functioning was evaluated by the Confusion, Hubbub and Order Scale (CHAOS; Matheny, Wachs, Ludwig, & Phillips, 1995).

#### Rationale for sample size

The primary outcome, to demonstrate feasibility, was recruitment into the trial and retention to follow‐up. We intended to recruit *N* = 100 participants to ensure 30 participants per group, conservatively assuming 35%–40% drop out (Jones et al., 2015), to reliably determine these outcomes.

### Data analysis

Sample characteristics including baseline mood and parenting variables, recruitment and retention levels, data completion and web usage were all summarised with descriptive statistics.

Clinical outcomes were analysed with R version 3.2.2 (R‐Core‐Team, 2015). Treatment effects through time were specified as piecewise linear at 16 weeks after randomisation (treatment endpoint). For HAM‐D and MAS, data were piecewise linear terms implemented at 24 weeks. Linear regressions with correlated errors, specifically by uniform correlation structure, based on exploratory data analyses and diagnostic checks, are used to assess these effects. Models were fitted using the R (R Core: R‐Core‐Team, 2015) package nlme (Pinheiro, Bates, DebRoy, Sarkar, & R‐Core‐Team, 2015). All analyses were run with and without controlling for baseline variables (parent gender, age, education and service contact, illness severity and history, family finances, family composition, child gender and age and presence of formal disabilities/health problems in child). As controlling for these variables did not impact on any results we have reported the former analyses for each clinical variable.

Differences in treatment arms in hazard for the first depression or mania data were analysed by Cox proportional hazards regression (Cox, 1972) using the R package survival (Therneau, 2015; Therneau & Grambsch, 2000). Missing data were assumed to be missing at random, based on inspecting the reasons for missing data, for which maximum likelihood would provide reliable results.

## Results

### Participant Characteristics (summarised by treatment arm in Table 2)

**Table 2:** Parent demographics at baseline

	WL group (*n* = 50)	IBPI group (*n* = 47)
Age in years, mean (*SD*)	36.3 (6.7)	37 (5.9)
Gender, female, *n* (%)	35 (70)	41 (87.2)
Age of bipolar diagnosis, mean (*SD*)	29.6 (7.5)	30 (6.7)
Diagnosis, *n* (%)		
Bipolar I	47 (94)	44 (93.6)
Bipolar II	3 (6)	3 (6.4)
Number of previous episodes, *n* (%)		
Depression		
1–6	9 (18)	8 (17)
>6	41 (82)	49 (83)
Mania/Hypomania		
1–6	10 (20)	16 (34)
>6	40 (80)	31 (66)
Depression (HRSD), *n* (%)		
0–13	42 (84)	39 (83)
>13	8 (16)	8 (17)
Mania (MAS), *n* (%)		
0–20	50 (100)	47 (100)
>20	0	0
Prescribed medication, *n* (%)		
Antidepressants	24 (48)	16 (34)
Mood Stabilisers	29 (58)	28 (60)
Antipsychotics	31 (62)	28 (60)
Service contact, *n* (%)		
General Practitioner	26 (52)	27 (57.4)
Care‐coordinator/CMHT	20 (40)	15 (31.9)
Psychiatrist	3 (6)	5 (10.6)
Psychologist/Therapist	1 (2)	0

CMHT, Community Mental Health Team; IBPI, Integrated Bipolar Parenting Intervention; WL, waitlist control.

Participants were <38 years of age, predominantly female (78%, *n* = 76) and ≤7 years since diagnosis of BD. Most (94%, *n* = 91) had a bipolar I diagnosis and subclinical scores on observer‐rated depression (84%, *n* = 81) and mania (100%, *n* = 97). Participants were prescribed a combination of antidepressants, mood stabilisers and antipsychotics. Most were receiving care from general practitioners (55%, *n* = 53) or care coordinators/community mental health teams (36%, *n* = 35). Supplementary demographic information concerning marital status, employment, family composition, ethnicity, schooling, and hospital admissions are in the Online Supplementary Material: Table S1. Data in Table 2 and Table S1 indicate both arms have similar characteristics.

### Feasibility outcomes

**Figure 1:**
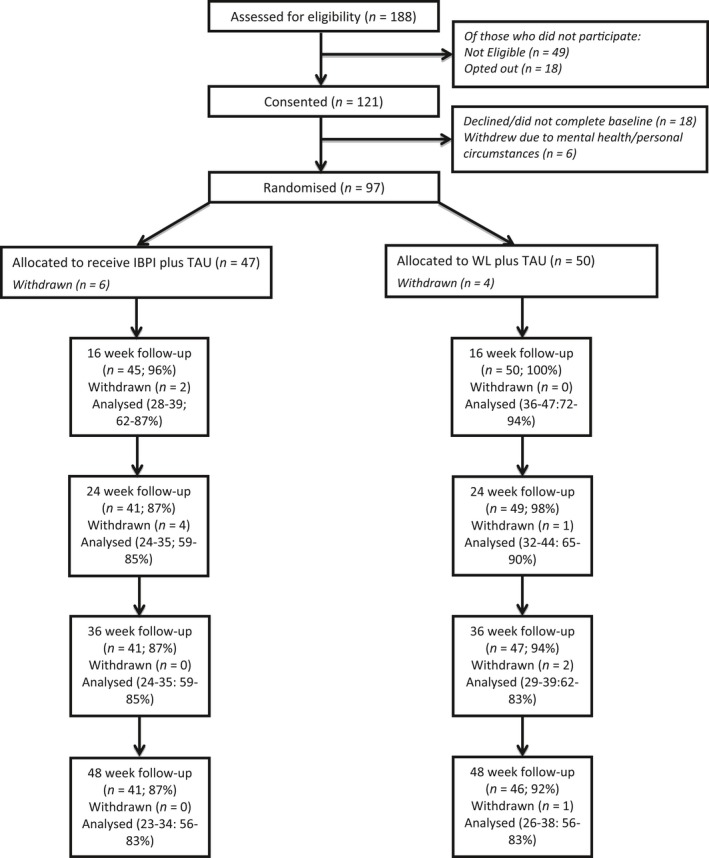
CONSORT diagram illustrating participant flow through the IBPI trial

One hundred and eighty‐eight individuals were screened for eligibility (Figure 1; 49 not eligible, 18 opted out), 121 consented (18 declined after consent, six withdrew due to mental health or personal issues). Ninety‐seven participants were randomised of whom 91% were retained (*n* = 88) to 36 weeks. Nine participants were due to have their 48 week assessment after end of the study period so received their final assessment including SCID‐LIFE, HAM‐D and MAS at 36 weeks. Of participants eligible to receive their 48 week assessment as planned, 89% (*n* = 78) were retained (total completed assessments, 90%; *n* = 87). Although retention was high, data for particular measures varied from 56% to 94% of those retained, range of retention (‘analysed’: number and percentage) across measures is summarised by arm and assessment point in Figure 1: highest completion rates were for observer‐rated measures, particularly HAM‐D and MAS (Figure 1, Table 3 and Table S3). Ten participants were lost following randomisation, one due to increased time commitments, two due to ill health and seven were not possible to contact further. No study‐related adverse events were recorded.

**Table 3:** Child behaviour and parenting measures

	WL group			IBPI group		
	*n*	Mean	*SD*	*n*	Mean	*SD*
Strengths and Difficulties Questionnaire						
Baseline	49	11.0	6.5	47	10.5	6.5
16 week follow‐up	47	12.6	7.1	39	8.6	5.6
24 week follow‐up	43	12.7	7.3	35	7.6	5.0
36 week follow‐up	39	12.4	8.4	35	7.4	5.5
48 week follow‐up	33	13.2	7.9	30	7.2	5.2
Eyberg Child Behavior Inventory – Problem						
Baseline	49	12.2	8.8	45	10.8	8.4
16 week follow‐up	44	11.5	7.9	32	8.8	8.7
24 week follow‐up	43	13.2	9.2	28	8.3	8.6
36 week follow‐up	35	11.6	9.5	28	7.5	5.7
48 week follow‐up	30	12.5	9.7	27	6.8	6.0
Eyberg Child Behavior Inventory – Intensity						
Baseline	49	117.5	38.5	45	115.3	34.1
16 week follow‐up	44	120.3	37.9	32	106.6	33.5
24 week follow‐up	43	121.6	35.2	28	105.9	28.0
36 week follow‐up	35	117.0	38.4	28	100.1	26.7
48 week follow‐up	29	114.7	41.8	27	98.2	27.9
Parenting Scale						
Baseline	48	3.3	0.6	45	3.2	0.6
16 week follow‐up	44	3.2	0.7	32	3.1	0.6
24 week follow‐up	43	3.2	0.6	28	3.1	0.5
36 week follow‐up	35	3.0	0.6	28	2.9	0.5
48 week follow‐up	30	3.0	0.7	27	3.1	0.6
Parenting Sense of Competency Scale						
Baseline	49	60.5	10.9	45	60.4	12.5
16 week follow‐up	43	59.6	12.4	32	64.9	9.4
24 week follow‐up	42	60.2	12.3	28	66.1	9.9
36 week follow‐up	34	62.6	13.8	28	65.4	10.6
48 week follow‐up	29	61.1	15.0	27	67.3	9.9
Parenting Stress Index						
Baseline	49	94.4	32.9	45	92.7	21.8
16 week follow‐up	44	94.3	23.9	32	82.5	18.9
24 week follow‐up	44	94.4	22.8	31	84.1	17.4
36 week follow‐up	35	84.9	24.8	28	81.9	18.9
48 week follow‐up	31	90.7	23.4	27	80.8	20.4

IBPI, Integrated Bipolar Parenting Intervention; WL, waitlist control.

#### Participant website use

Of 47 participants randomised to IBPI, 77% (*n* = 36) accessed bipolar modules (Table S2). Module 1 (What is Bipolar Disorder?) was visited by the most (92%, *n* = 33), module 8 (Finding support and final thoughts) the least (14%, *n* = 5). Average number of visits per person ranged from ~10 for modules 1–3 (1; mean ($\overset{¯}{x}$) = 9.97, standard deviation (*SD*) = 8.05: 2; $\overset{¯}{x}$ = 9.95, *SD* = 7.0; 3; $\overset{¯}{x}$ = 10.11, *SD* = 4.03) to $\overset{¯}{x}$ = 5.2, *SD* = 2.38 for module 8.

Twenty‐five participants (53%) accessed Triple P modules. All parents visited module 1 (What is positive parenting?: 100%, *n* = 25) whereas only five visited modules 5–8 respectively (Dealing with disobedience; Preventing problems by planning ahead; Making shopping fun; Raising confident, competent kids: 20%). Average number of visits per person ranged from $\overset{¯}{x}$ = 4.20 (*SD* = 2.52) for module 1 to $\overset{¯}{x}$ = 1.4 (*SD* = 0.55) for module 8.

#### Baseline score for parenting and child behaviour

Child behaviour problems on SDQ were approaching borderline abnormal behaviour cut‐off (Goodman, 2001; Table 3). For ECBI, number of problems (ECBI‐P) and intensity (ECBI‐I) were elevated (Colvin, Eyeberg & Adams, 1999). Dysfunctional parenting (PS3) was at clinical levels (Arnold et al., 1993). Parenting confidence (PSOC) was moderate and parenting stress levels (PSI) were clinically significant (Abidin, 1990; Johnston & Mash, 1989).

#### Baseline scores for self‐rated parental mood and family coherence

Internal States Scale‐Activation scores and Altman Scales were below mania cut‐offs (Altman et al., 1997; Bauer et al., 1991; Table S3). ISS Wellbeing did not indicate depression, although CES‐D scores did (Radloff, 1991). Household disturbance (CHOAS) was extremely high compared to a general population sample (Coldwell, Pike, & Dunn, 2006). Scores were similar in both arms.

### Primary clinical outcomes (Tables 3 and 4; Figures S1–S6)

**Table 4:** Comparison of change in child behaviour and parenting and mood measures IBPI versus WL

	Difference in slopes during intervention				Difference in slopes post intervention			
	Est.	*SE*	*CI*	*p*	Est.	*SE*	*CI*	*p*
Child Behaviour								
SDQ	0.83	0.19	0.45, 1.20	<.01	−0.11	0.12	−0.34, 0.12	.34
ECBI‐P	0.34	0.30	−0.25, 0.93	.26	0.14	0.19	−0.24, 0.51	.47
ECBI‐I	1.43	1.07	−0.66, 3.53	.18	0.16	0.66	−1.14, 1.45	.81
Parenting								
PS3	0.03	0.02	−0.02, 0.07	.24	−0.02	0.01	−0.05, 0.01	.14
PSOC	−1.17	0.41	−1.98, −0.37	<.01	−0.01	0.26	−0.53, 0.50	.96
PSI	1.94	0.77	0.42, 3.45	.01	−0.90	0.49	−1.85, 0.06	.06

ECBI‐I, Eyberg Child Behavior Inventory Intensity; ECBI‐P, Eyberg Child Behavior Inventory Problem; IBPI, Integrated Bipolar Parenting Intervention; PS3, Parenting Scale; PSI, Parenting Stress Index; PSOC, Parenting Sense of Competency Scale; SDQ, Strengths and Difficulties Questionnaire; WL, waitlist control.

#### Child behaviour

Strengths and Difficulties Questionnaire improved significantly to end of treatment in IBPI (−0.48 units per 4 weeks, 95%, CI −0.76 to −0.19, *SE* = 0.14, *p *<* *.01) but worsened in WL (0.35 units per 4 weeks, 95% CI 0.09 to 0.61, *SE* = 0.13, *p *<* *.01): this slope difference was significant. After 16 weeks SDQ did not change (IBPI; 0.01 units per 4 weeks, 95% CI −0.15 to 0.18, *SE* = 0.09, *p *<* *.87: WL; −0.10 units per 4 weeks; 95% CI −0.26 to 0.06, *SE* = 0.08, *p *<* *.23) indicating persistence of gains in IBPI.

ECBI‐P did not change significantly for either group during intervention (IBPI; −0.30 units per 4 weeks; 95% CI −0.77 to 0.17, *SE* = 0.24, *p *<* *.24: WL; 0.04 units per 4 weeks; 0.06, 95% CI −0.36 to 0.48, *SE* = 0.21, *p *<* *.7) or follow‐up (IBPI; −0.15 units per 4 weeks; 95% CI −0.43 to 0.13, *SE* = 0.14, *p *<* *.29: WL; −0.01 units per 4 weeks; 95% CI −0.28 to 0.24, *SE* = 0.13, *p *<* *.94).

ECBI‐I indicated the same pattern during intervention (IBPI; 0.79 units per 4 weeks; 95% CI −2.42 to 0.84, *SE* = 0.83, *p *<* *.24: WL; 0.64 units per 4 weeks; 95% CI −0.75 to 2.04, *SE* = 0.71, *p *<* *.37) and follow‐up (IBPI; −0.61 units per 4 weeks; 95% CI −1.57 to 0.34, *SE* = 0.49, *p *<* *.21: WL; −0.46 units per 4 weeks; 95% CI −1.32 to 0.42, *SE* = 0.45, *p *<* *.31). Slopes differences were not statistically significant.

#### Parenting skills and confidence

During intervention, dysfunctional parenting (PS3) decreased significantly for IBPI (−0.04 units per 4 weeks, 95% CI −0.08 to −0.01, (.E. 0.02, *p *<* *.02) but not WL (−0.02 units per 4 weeks, 95% CI −0.05 to 0.01, *SE* = 0.02, *p *<* *.27). Difference in slopes was not significant. After 16 weeks PS3 did not change in IBPI (−0.004 units per 4 weeks, 95% CI −0.03 to 0.02, *SE* = 0.01, *p *<* *.68) but decreased in WL (0.03 units per 4 weeks; 95% CI −0.05 to −0.01, *SE* = 0.01, *p *<* *.01). Difference in slopes was not significant.

Parenting confidence (PSOC) increased significantly for IBPI (1.18 units per 4 weeks, 95% CI 0.55 to 1.82, *SE* = 0.32, *p *<* *.01) but not for WL to 16 weeks (0.01 units per 4 weeks, 95% CI −0.53 to 0.56, *SE* = 0.28, *p *<* *.96). This difference in slopes was significant. After 16 weeks PSOC did not change in either arm indicating persistence of gains for IBPI (IBPI; 0.19 units per 4 weeks, 95% CI −0.18 to 0.57, *SE* = 0.19, *p *<* *.32: WL 0.18 units per 4 weeks; 95% CI −0.17 to 0.53, *SE* = 0.18, *p *<* *.31).

Parenting stress (PSI) reduced significantly during treatment for IBPI (−1.87 units per 4 weeks, 95% CI −3.07 to −0.68, *SE* = 0.61, *p *<* *.01) but not WL (0.06 units per 4 weeks, 95% CI −0.96 to 1.08, *SE* = 0.52, *p *<* *.91). This difference in slopes was significant. After intervention there was no change in IBPI (0.02 units per 4 weeks, 95% CI −0.69 to 0.72, *SE* = 0.36, *p *<* *.96) but a significant decrease in WL (−0.88 units per 4 weeks, 95% CI −1.51 to 0.24, *SE* = 0.32, *p *<* *.01). Difference in slopes was not significant.

### Secondary clinical outcomes

No significant differences were observed for secondary clinical outcomes. Table S3 provides summary data for family functioning, self and observed‐rated mood, Table S4 indicates comparative analysis of slopes. Figures S7–S9 illustrate time to relapse outcomes for any mood episode, mania and depression. These did not differ between groups.

## Discussion

IBPI is a new online intervention for parents with BD with young children, combining self‐management information about BD with parenting information in an interactive format and is the only online intervention of this type we are aware of. This study explored the feasibility and acceptability of IBPI and the trial design and provided preliminary estimates of clinical effects. Retention rate was 90% to 48 weeks follow‐up balanced across both arms (analysable data varied across measures). 75% of participants accessed the bipolar self‐help modules and 53% accessed Triple P modules: earlier modules were more popular for both. The low level of access to parenting modules is of concern for an intervention intended to improve parenting outcomes. Future iterations of this intervention could explore both improved integration of bipolar and Triple P modules and possibly whether parenting modules alone improve uptake. It is acknowledged that this sample was self‐selected which might reduce generalisability of our findings, for instance most of our sample were educated to degree level.

Despite modest level of use for Triple P content in particular, clinical outcomes suggest IBPI participants improved relative to WL. Child behaviour problems (SDQ) improved significantly during access to IBPI sustained throughout follow‐up. A similar but nonsignificant pattern was observed for ECBI problems and intensity. Parenting stress and confidence (PSI and PSOC) improved significantly during the intervention, sustained through follow‐up. Dysfunctional parenting (PS3) also improved during IBPI but more marginally as difference in slopes was not significant. Household disturbance (CHAOS) did not change in either arm. These results were despite parenting problems not being a selection criterion. It is possible that greater differences between groups might have been observed had this criterion been included and this should be considered in a future trial.

Parental mood and relapse were not significantly different between arms although numerical patterns favoured IBPI. An appropriately powered trial is required to evaluate definitively whether improvements in child behaviour and parenting have secondary benefits for parental mood and relapse in a broader sample.

There have been few studies of online parenting interventions for severe mental health problems and only one for BD specifically (Jones et al., 2014). IBPI is less resource intensive than approaches described in other online interventions (Danaher et al., 2013; Kaplan et al., 2014; O'Mahen et al., 2014; van der Zanden, Speetjens, Arntz, & Onrust, 2010), as it did not include a facilitated forum or chat sessions. Our findings are consistent with outcomes of studies of Triple P Online with healthy parents of children with disruptive behaviour (Sanders et al., 2012; Sanders, Dittman et al., 2014).

This study has a number of limitations. Firstly, it was designed primarily to determine feasibility and acceptability so was not powered specifically to detect clinical outcome. Despite this there was significant and sustained change in some parenting and child behaviour measures, but other effects may have been missed due to power issues. Secondly, although BD and parenting elements of IBPI were designed to be linked, integration was not perfect as Triple P Online is designed for parents of all types. Stronger results might have been obtained from a further iteration of the intervention to tailor the parenting modules more specifically to BD. Thirdly, although overall retention was high, there was variability in the number of measures completed by participants at each time point. Exploration of additional approaches to enhance data completeness would be important for a future definitive trial. Highest completion rates were for observer‐rated measures so consideration should be given to providing telephone support for self‐report measures. Fourthly, level of site use was quite low, Sanders et al. (2012) reported that 96% of their parents used Triple P Online compared with 53%, in the current study (77% accessed the bipolar modules). Future studies should incorporate factors likely to further enhance site engagement including additional e‐prompts (Alkhaldi et al., 2016).

## Conclusions

IBPI is a feasible intervention with positive outcomes for parenting and child behaviour. A future definitive trial is needed to confirm current effects, explore possible impact on parental mood and determine cost‐effectiveness. IBPI has potential to deliver an accessible, nonstigmatising intervention to bipolar parents at low cost. As this intervention requires very little professional support it could be offered as a supplement to current services without significant additional investment. Further research will be needed to explore in the longer term whether the beneficial impacts of the intervention translate into reduced risks of longer‐term mental health problems in addition to shorter term improvements in current child behaviour.

**Key points**

- The study compared a novel web‐based self‐management intervention for parents with bipolar disorder with children aged 3–10 years with waitlist control in an RCT.
- The intervention and trial design were feasible and acceptable.
- Parents in the intervention arm reported improved child behaviour and parenting to 48 week follow‐up.
- Parental mood and mood relapses were not significantly different between treatment arms.
- This low cost, flexible intervention may have potential for supporting parents with bipolar disorder.
- Longer term benefits for children are unknown.

## Supporting information


**Table S1.** Supplementary demographic information.
**Table S2.** Modules visits to IBPI.
**Table S3.** Parent Mood Measures.
**Table S4.** Comparison of change in parent mood measures IBPI vs. WL.
**Figure S1.** Strengths and Difficulties Questionnaire (SDQ) Scores as a Function of Group.
**Figure S2.** Eyberg Child Behavior Inventory Problem (ECBI‐P) Scores as a Function of Group.
**Figure S3.** Eyberg Child Behavior Inventory Intensity (ECBI‐I) Scores as a Function of Group.
**Figure S4.** Parenting Scale (PS3) Scores as a Function of Group.
**Figure S5.** Parenting Sense of Competency (PSOC) Scores as a Function of Group.
**Figure S6.** Parenting Stress Index (PSI) Scores as a Function of Group.
**Figure S7.** Kaplan–Meier estimates of time to first depressive or manic recurrence over 48 weeks follow‐up.
**Figure S8.** Kaplan–Meier estimates of time to first depressive recurrence over up to 48 weeks follow‐up.
**Figure S9.** Kaplan–Meier estimates of time to first manic recurrence over up to 48 weeks follow‐up.

**Figure S10.** CONSORT 2010 checklist.
