# Supplementary material for: Web‐based integrated bipolar parenting intervention for parents with bipolar disorder: a randomised controlled pilot trial
Source: J Child Psychol Psychiatry. 2017 May 16;58(9):1033–41. doi: 10.1111/jcpp.12745 (PMC5573909; doi:10.1111/jcpp.12745)
Supplement: Supplementary file 1 — Table S1. Supplementary demographic information. Table S2. Modules visits to IBPI. Table S3. Parent Mood Measures. Table S4. Comparison of change in parent mood measures IBPI vs. WL. Figure S1. Strengths and Difficulties Questionnaire (SDQ) Scores as a Function of Group. Figure S2. Eyberg Child Behavior Inventory Problem (ECBI‐P) Scores as a Function of Group. Figure S3. Eyberg Child Behavior Inventory Intensity (ECBI‐I) Scores as a Function of Group. Figure S4. Parenting Scale (PS3) Scores as a Function of Group. Figure S5. Parenting Sense of Competency (PSOC) Scores as a Function of Group. Figure S6. Parenting Stress Index (PSI) Scores as a Function of Group. Figure S7. Kaplan–Meier estimates of time to first depressive or manic recurrence over 48 weeks follow‐up. Figure S8. Kaplan–Meier estimates of time to first depressive recurrence over up to 48 weeks follow‐up. Figure S9. Kaplan–Meier estimates of time to first manic recurrence over up to 48 weeks follow‐up. [file JCPP-58-1033-s001.docx]

**Online supplementary material for - Web-based integrated bipolar parenting intervention (IBPI) for parents with bipolar disorder: A randomised controlled pilot trial - by Jones et al.**

**Table S1:** Supplementary demographic information

|  |  |  |  |
| --- | --- | --- | --- |
|  | | WL group  (n = 50) | IBPI group  (n = 47) |
| Marital Status, n (%) | |  |  |
| Married | | 22 (44) | 27 (57.4) |
| Divorced / Separated | | 12 (24) | 7 (14.9) |
| Single | | 8 (16) | 7 (14.9) |
| Cohabiting | | 7 (14) | 5 (10.6) |
| Widow / Widower | | 1 (2) | 0 |
| Civil partnership | | 0 | 1 (2.1) |
| Employment Status, n (%) | |  |  |
| Full-time | | 10 (20) | 9 (19.1)) |
| Part-time | | 8 (16) | 17 (36.2) |
| Voluntary | | 0 | 1 (2.1) |
| Self-employed | | 0 | 4 (8.5) |
| Retired | | 1 (2) | 0 |
| Student | | 3 (6) | 0 |
| Sick / disability | | 7 (14) | 3 (6.4) |
| Unemployed | | 21 (42) | 13 (27.7) |
| Family Composition, n (%) | |  |  |
| Original family | | 25 (50) | 27 (57.4) |
| Step-family | | 6 (12) | 5 (10.6) |
| Sole-parent family | | 18 (36) | 14 (29.8) |
| Other | | 1 (2) | 1 (2.1) |
| Ethnicity, n (%) | |  |  |
| Pakistani | | 1 (2) | 2 (4.3) |
| Other mixed background | | 1 (2) | 0 |
| White British | | 48 (96) | 41 (87.2) |
| White Irish | | 0 | 1 (2.1) |
| Other white background | | 0 | 2 (4.3)_ |
| Other ethnic group | | 0 | 1 (2.1) |
| Education, n (%) | |  |  |
| GSCEs or equivalent completed | | 9 (18) | 8 (17) |
| Further education – not completed | | 1 (2) | 1 (2) |
| Further education – completed | | 18 (36) | 11 (23) |
| Higher education – not completed | | 2 (4) | 2 (4) |
| Higher education – completed | | 13 (26) | 16 (34) |
| Postgraduate or equivalent – not completed | | 0 | 0 |
| Postgraduate or equivalent – completed | | 7 (14) | 9 (19) |
| Hospital Admissions, n (%) | |  |  |
| 0-6 | | 45 (90) | 44 (94) |
| >6 | | 5 (10) | 3 (6) |

**Table S2**: Modules visits to IBPI

| Module | Number and percentage of parents  (n=47) | Number of visits per parent | |
| --- | --- | --- | --- |
|  |  | Mean | SD |
| Bipolar |  | | |
| 1. What is Bipolar Disorder? | 33 (70) | 9.97 | 8.06 |
| 2. Benefits and Challenges | 22 (46) | 9.95 | 7.06 |
| 3. Managing Emotions | 17 (36) | 10.11 | 4.03 |
| 4. Knowing Yourself | 13 (28) | 10.54 | 5.78 |
| 5. Mood Monitoring | 16 (34) | 10.19 | 9.28 |
| 6. Playing to your strengths | 6 (13) | 8.17 | 7.13 |
| 7. Planning for yourself | 7 (15) | 5.57 | 4.79 |
| 8. Finding support and final thoughts | 5 (11) | 5.20 | 2.39 |
| Triple P Online |  | | |
| 1. What is positive parenting? | 25 (53) | 4.20 | 2.52 |
| 2.Encouraging desirable behaviours | 18 (38) | 1.89 | 0.76 |
| 3. Teaching new skills and behaviour | 15 (32) | 2.47 | 1.41 |
| 4. Managing misbehaviour | 7 (15) | 1.57 | 0.79 |
| 5. Dealing with disobedience | 5 (11) | 1.60 | 0.50 |
| 6. Preventing problems by planning ahead | 5 (11) | 1.60 | 1.00 |
| 7. Making shopping fun | 5 (11) | 1.80 | 0.82 |
| 8. Raising confident, competent kids | 5 (11) | 1.40 | 0.55 |

**Table S3:** Parent Mood Measures

|  | WL group | | | IBPI group | | |
| --- | --- | --- | --- | --- | --- | --- |
|  | n | Mean | s.d. | n | Mean | s.d. |
| Internal States Scale - Activation |  |  |  |  |  |  |
| Baseline | 43 | 149 | 129 | 42 | 110 | 127 |
| 16 week follow-up | 38 | 167 | 139 | 32 | 96 | 95 |
| 24 week follow-up | 37 | 129 | 118 | 25 | 132 | 113 |
| 36 week follow-up | 34 | 121 | 115 | 28 | 133 | 128 |
| 48 week follow-up | 29 | 126 | 122 | 27 | 105 | 101 |
| Internal States Scale - Wellbeing |  |  |  |  |  |  |
| Baseline | 44 | 150 | 74 | 44 | 124 | 79 |
| 16 week follow-up | 42 | 125 | 77 | 32 | 129 | 77 |
| 24 week follow-up | 40 | 136 | 82 | 27 | 162 | 80 |
| 36 week follow-up | 34 | 143 | 84 | 28 | 148 | 72 |
| 48 week follow-up | 30 | 117 | 87 | 27 | 121 | 57 |
| Centre for Epidemiologic Studies Depression Scale | |  |  |  |  |  |
| Baseline | 42 | 22.4 | 134 | 36 | 18.6 | 11.6 |
| 16 week follow-up | 36 | 26.0 | 140 | 29 | 23.1 | 15.1 |
| 24 week follow-up | 32 | 23.7 | 170 | 24 | 22.0 | 15.5 |
| 36 week follow-up | 32 | 20.4 | 162 | 24 | 23.0 | 15.8 |
| 48 week follow-up | 26 | 23.4 | 186 | 23 | 23.3 | 15.8 |
| Altman Self-Rating Mania Scale |  |  |  |  |  |  |
| Baseline | 46 | 4.9 | 4.4 | 44 | 5.7 | 5.0 |
| 16 week follow-up | 38 | 4.4 | 4.1 | 28 | 2.4 | 2.5 |
| 24 week follow-up | 40 | 4.2 | 4.4 | 25 | 5.0 | 3.2 |
| 36 week follow-up | 29 | 3.7 | 3.9 | 26 | 3.7 | 3.6 |
| 48 week follow-up | 29 | 2.8 | 2.9 | 27 | 3.7 | 3.3 |
| Confusion, Hubbub and Order Scale |  |  |  |  |  |  |
| Baseline | 49 | 14.8 | 4.0 | 45 | 15.1 | 3.9 |
| 16 week follow-up | 41 | 15.5 | 4.5 | 30 | 15.2 | 3.8 |
| 24 week follow-up | 42 | 15.6 | 4.3 | 27 | 14.0 | 3.4 |
| 36 week follow-up | 35 | 15.3 | 3.9 | 27 | 14.1 | 3.5 |
| 48 week follow-up | 30 | 15.2 | 3.8 | 26 | 14.9 | 3.9 |
| Hamilton Depression Rating Scale |  |  |  |  |  |  |
| Baseline | 50 | 7.1 | 7.1 | 47 | 6.6 | 6.2 |
| 24 week follow-up | 44 | 6.3 | 6.5 | 35 | 5.3 | 4.5 |
| 48 week follow-up | 38 | 7.0 | 7.8 | 34 | 6.8 | 5.9 |
| Bech-Rafaelsen Mania Scale |  |  |  |  |  |  |
| Baseline | 50 | 3.5 | 4.4 | 47 | 3.5 | 3.5 |
| 24 week follow-up | 44 | 3.2 | 3.2 | 35 | 2.7 | 3.9 |
| 48 week follow-up | 38 | 2.2 | 2.5 | 34 | 2.2 | 2.7 |
|  |  |  |  |  |  |  |

**Table S4:** Comparison of change in parent mood measures IBPI vs. WL

|  | Difference in slopes during intervention | | | | Difference in slopes post intervention | | | |
| --- | --- | --- | --- | --- | --- | --- | --- | --- |
|  | Est. | *SE* | *CI* | *p* | Est. | *SE* | *CI* | *p* |
| Family Functioning |  |  |  |  |  |  |  |  |
| CHAOS | 0.13 | 0.15 | -0.16, 0.42 | 0.37 | 0.05 | 0.10 | -0.14, 0.23 | 0.62 |
| Parental Mood |  |  |  |  |  |  |  |  |
| ISS-A | 0.78 | 0.57 | -0.34, 1.89 | 0.17 | -0.74 | 0.40 | -1.53, 0.04 | 0.06 |
| ISS-W | -0.39 | 0.38 | -1.14, 0.35 | 0.30 | 0.07 | 0.29 | -0.51, 0.64 | 0.82 |
| CES-D | 0.45 | 0.76 | -1.04, 1.95 | 0.55 | -0.57 | 0.57 | -1.68, 0.54 | 0.31 |
| ASRM | 0.35 | 0.20 | -0.04, 0.73 | 0.08 | -0.26 | 0.14 | -0.54, 0.02 | 0.07 |
| MAS | 0.08 | 0.12 | -0.16, 0.32 | 0.53 | -0.06 | 0.15 | -0.36, 0.25 | 0.71 |
| HAM-D | 0.17 | 0.24 | -0.29, 0.64 | 0.46 | -0.16 | 0.31 | -0.76, 0.44 | 0.60 |

ASRM, Altman Mania Rating Scale; CES-D, Centre for Epidemiologic Studies Depression Scale; CHAOS, Confusion, Hubbub and Order Scale; HAM-D, Hamilton Depression Rating Scale; ISS-A, Internal States Scale Activation; ISS-W, Internal States Scale Wellbeing; MAS, Bech-Rafaelsen Mania Scale

**Figure S1:** Strengths and Difficulties Questionnaire (SDQ) Scores as a Function of Group

**
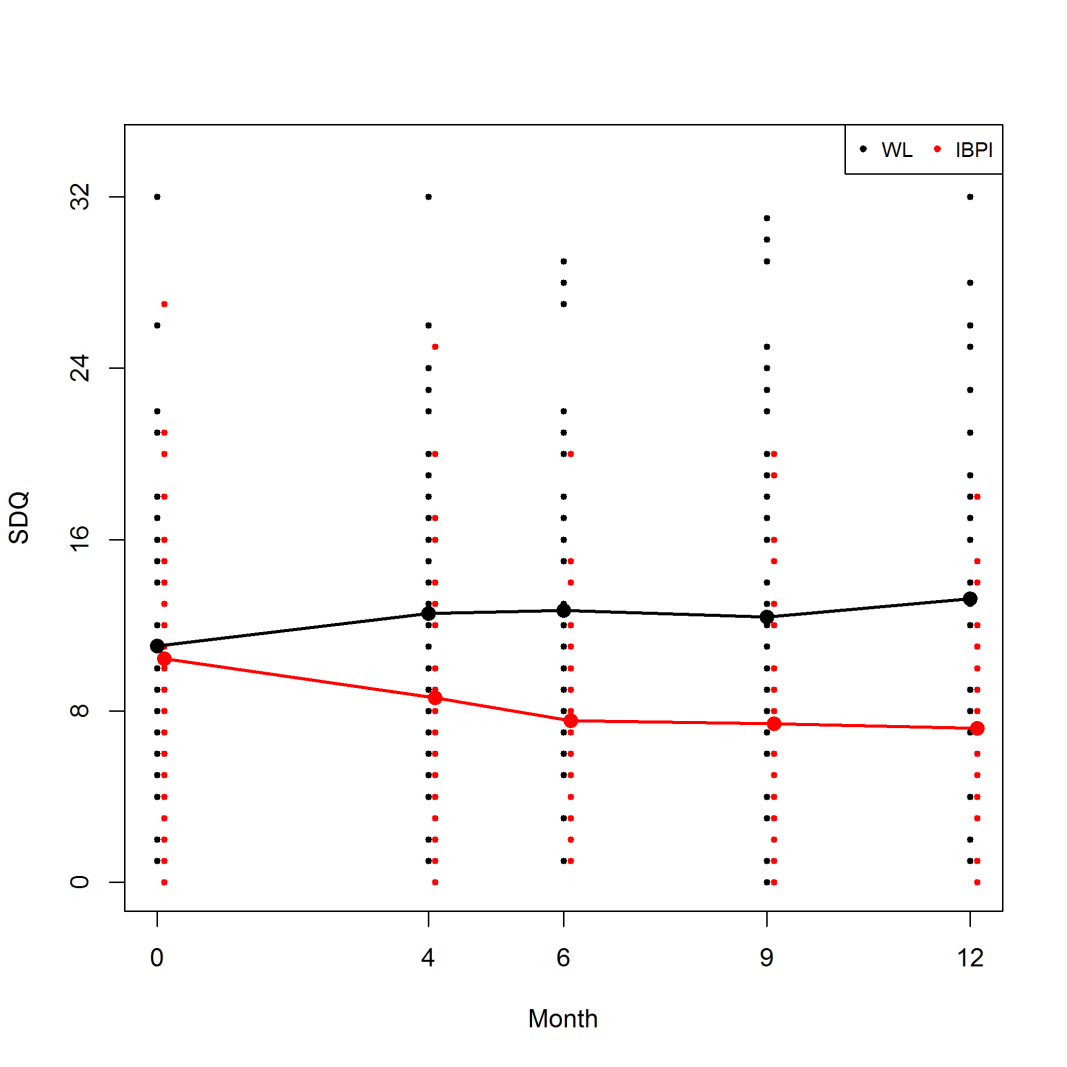
**

**Figure S2:** Eyberg Child Behavior Inventory Problem (ECBI-P) Scores as a Function of Group

**
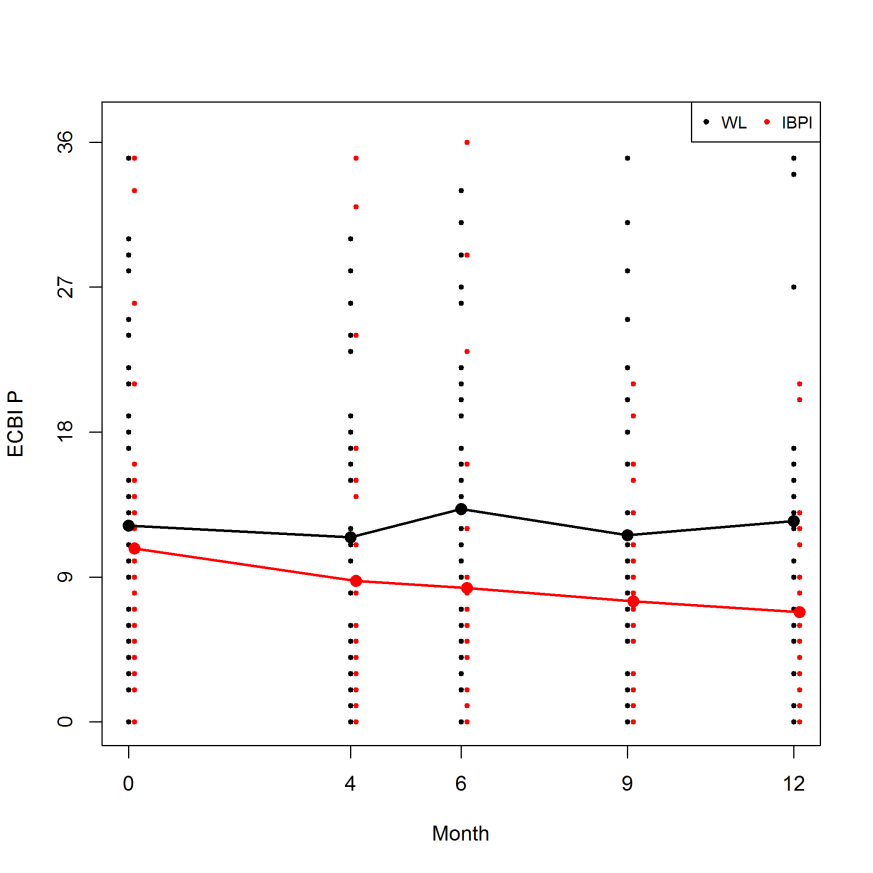
**

**Figure S3:** Eyberg Child Behavior Inventory Intensity (ECBI-I) Scores as a Function of Group

**
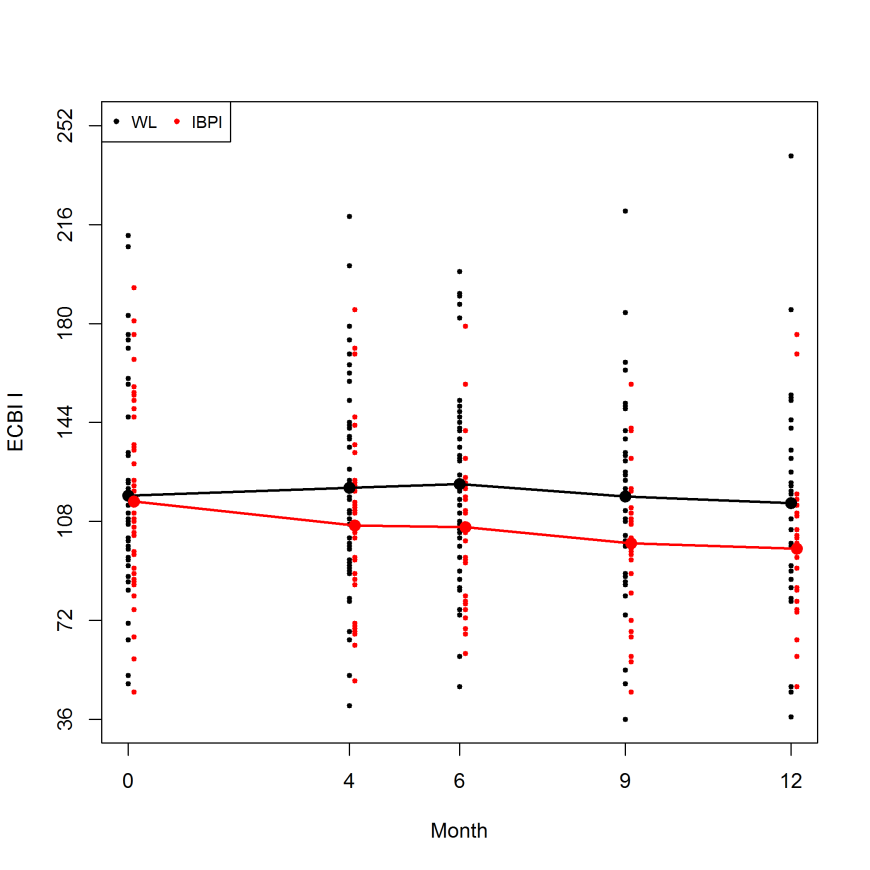
**

**Figure S4:** Parenting Scale (PS3) Scores as a Function of Group

**
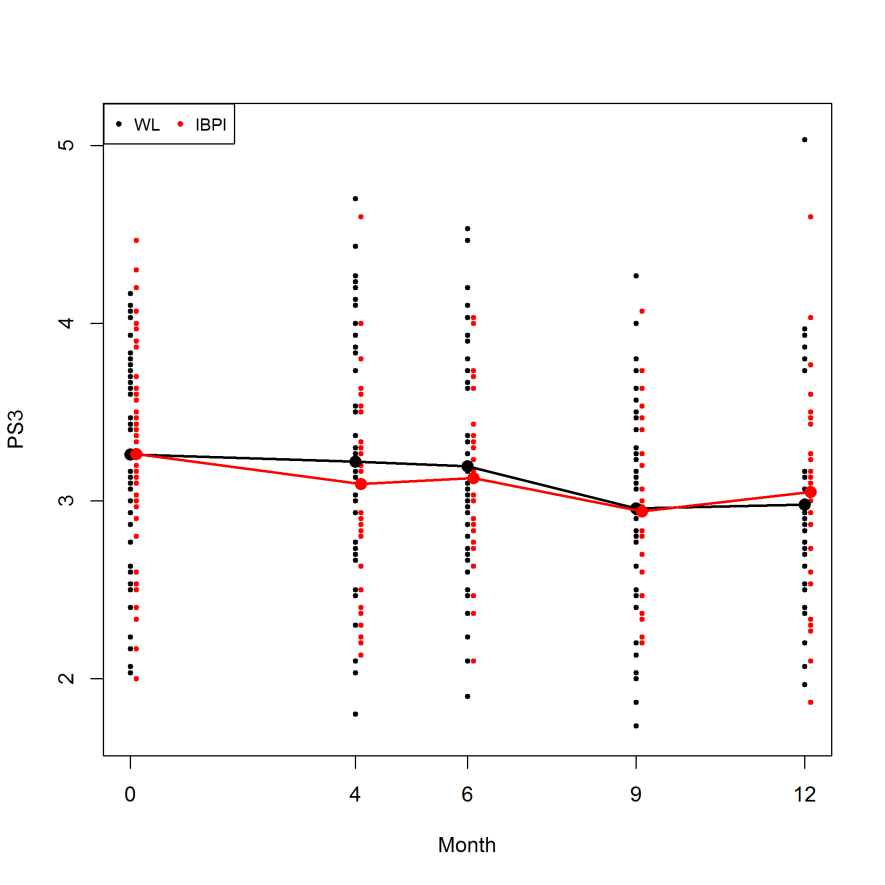
**

**Figure S5:** Parenting Sense of Competency (PSOC) Scores as a Function of Group

**
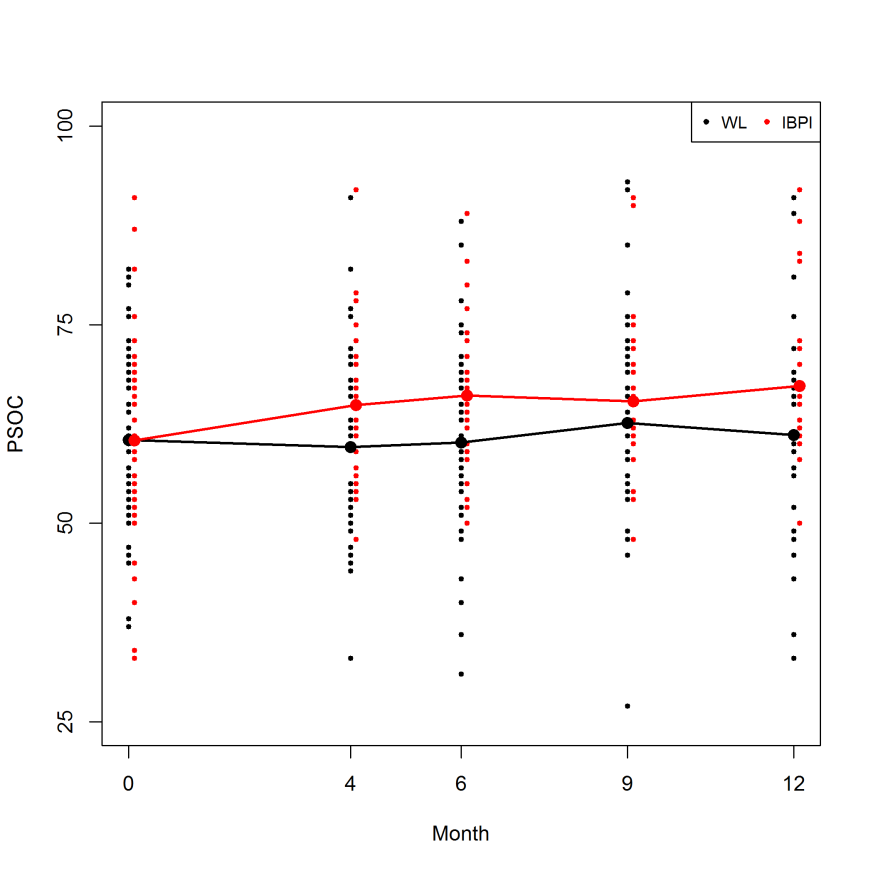
**

**Figure S6:** Parenting Stress Index (PSI) Scores as a Function of Group

**
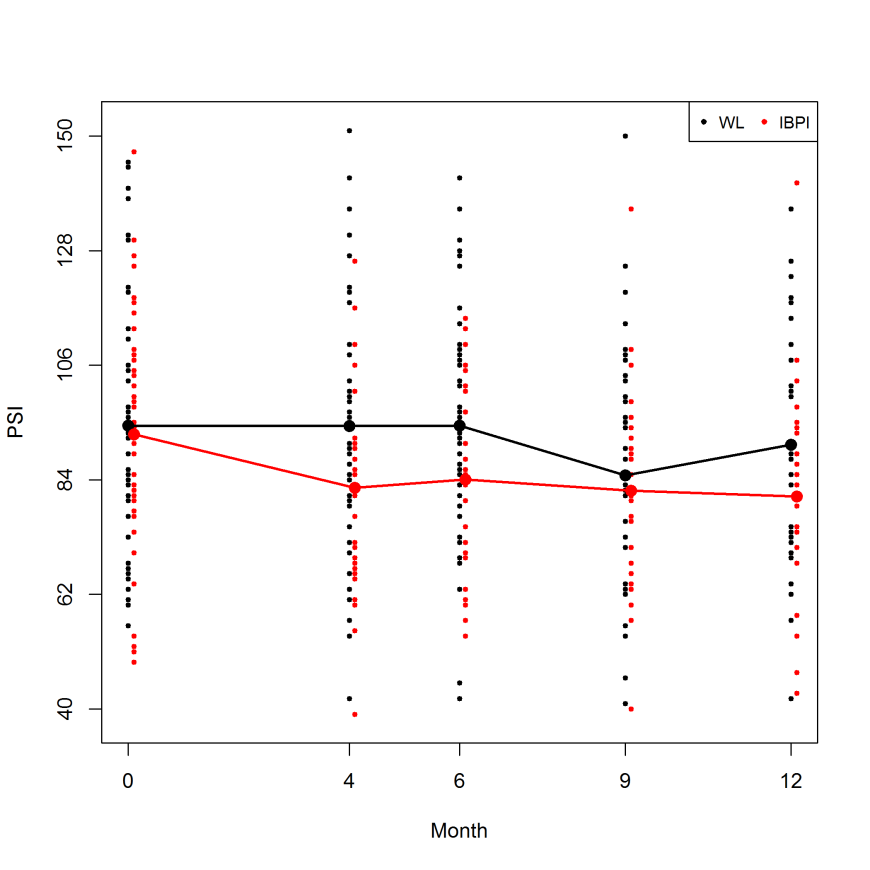
**

**Figure S7:** Kaplan–Meier estimates of time to first depressive or manic recurrence over 48 weeks follow-up.

**
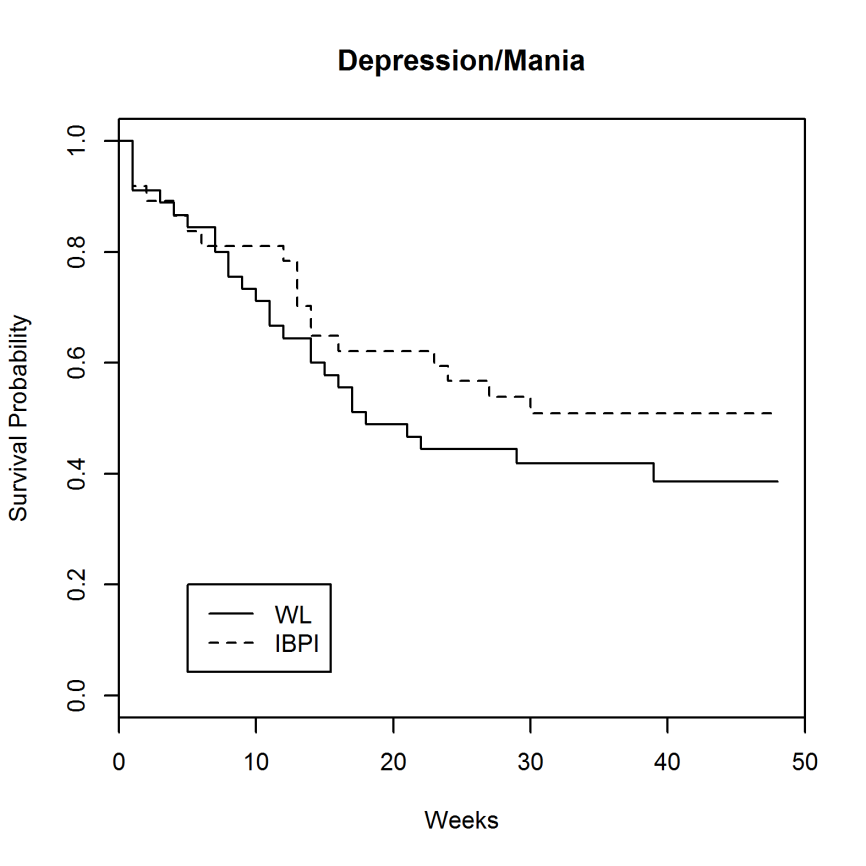
**

Estimated HR = 0.89 (95% CI: 0.38 – 2.06), SE = 0.43, Z = -0.27, *p* = 0.78 (based on Cox proportional hazards regression)

**Figure S8:** Kaplan–Meier estimates of time to first depressive recurrence over up to 48 weeks follow-up.

**
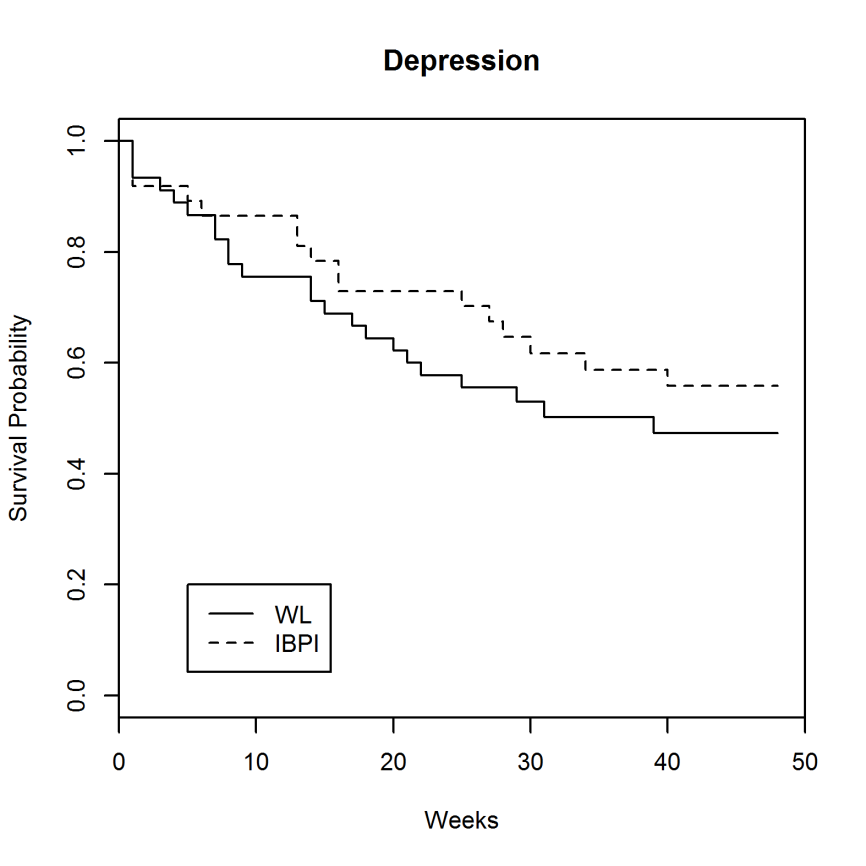
**

Estimated HR = 0.75 (95% CI: 0.39 – 1.41), SE = 0.33, Z = -0.90, *p* = 0.37 (based on Cox proportional hazards regression)

**Figure S9:** Kaplan–Meier estimates of time to first manic recurrence over up to 48 weeks follow-up.


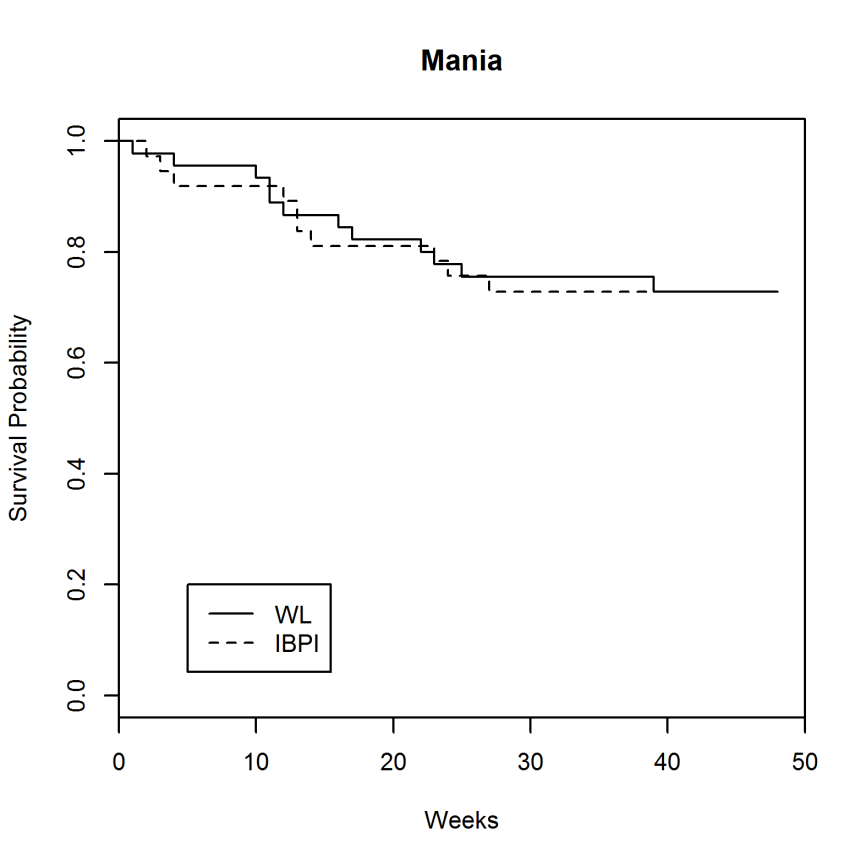


Estimated HR = 1.01 (95% CI: 0.44 – 2.35), SE = 0.43, Z = 0.03, *p* = 0.97 (based on Cox proportional hazards regression)
